# Thoraco-abdominal impalement injury with two construction iron bars – A rare case report

**DOI:** 10.1016/j.ijscr.2020.01.030

**Published:** 2020-01-27

**Authors:** Jitendra Sankpal, Kushagra Rahul, Aditya Phadke, Sushrut Sankpal

**Affiliations:** a General Surgery, Grant Government Medical College & Sir JJ Group of Hospitals, Mumbai, India; b Rajiv Gandhi Medical College, Thane, Mumbai, India

**Keywords:** Thoracoabdominal impalement injury, Rod injury, Construction bar injury

## Abstract

**Introduction:**

The work has been reported in line with the SCARE criteria. Thoracoabdominal impalement injuries are uncommon and very few cases have been reported. Impalement injuries result when a rigid object penetrates and remains lodged within the body. It has complex anesthetic and surgical management. We describe the successful surgical and anesthetic management of a major impalement injury of the torso.

**Case Report:**

A 21-year old male construction worker brought to emergency with two iron construction rods impaled in torso due to fall from 2nd floor while working. Both were 1 m long and 12 mm in diameter. One had penetrated from right anterior axillary fold, deep to pectoralis major, exiting from left sternal border. Second entered below the tip of right scapula and exiting from left of xiphoid process. ATLS protocols were followed and patient resuscitated, immediately shifted to operating room, intubated in semi left lateral position. Rod impacted in right pectoral area was superficial with no injury to ribs or pleural space. Other was removed through laparotomy, thoracotomy and Hepatotomy, as it had pierced diaphragm and liver. Post-operative recovery was uneventful.

**Discussion:**

Resuscitation and close monitoring prior to and during surgery are vital with anticipation of major organ and vascular injuries. Hypovolemia should be corrected in the OR. Progressive dyspnea can be the most important symptom in patients with penetrating chest injury.

**Conclusion:**

Penetrating abdomino-thoracic injuries demand immediate life-saving measures, appropriate resuscitative care, urgent shifting of patient to tertiary care center, prompt diagnosis and immediate surgical intervention. Regulation of safety rules at construction site and early intervention in case of accidents can improve the patient outcome and minimize mortality.

## 1 Introduction

The work has been reported in line with the SCARE criteria [6]. Thoracoabdominal impalement injuries are relatively uncommon and only a few cases have been reported in the literature. They are potentially life threatening due to the associated hemorrhagic shock and visceral injury. While many patients die at the scene, they often produce complex anesthetic and surgical challenge due to the inability in positioning and transport of the patient and the risk of sudden hemorrhage [1]. A great deal of force is required to impale thorax and thus there is extensive local tissue destruction with elements of both blunt and penetrating injury. Management of such cases provides a challenge to anesthetists and surgeons as the extent of injury is unknown and there is inadequate time for evaluation and resuscitation of the patient. Written informed consent was obtained from the patient for publication of this case report and any accompanying images.

## 2 Case report

A 21-year old male construction worker was brought to casualty department with two 12 mm iron bars/rods (used for construction) impaled through his torso. He had fall from second floor slab of under construction building and landed on 3-meter long, two 12 mm iron rods projecting from column. Fire brigade rescue team showing presence of mind had carefully used rod cutter to cut these rods to rescue and evacuate the patient. He was transported in sitting position in ambulance within one hour of fall. In casualty he was conscious and comfortable in sitting position, hemodynamically stable but in excruciating pain. Both the entry wound had impacted piece of cloth of his shirt. First 12 mm iron bar was 1 m long, and had penetrated from right anterior axillary fold just below the level of nipple, traversing pectoralis major muscle and exiting out from left sternal border at the level of third costo-chondral joint. Second 12 mm iron bar was 1 m long, and had penetrated just below the tip of right scapula and exiting out from below left of xiphoid process. None of blood or radiological investigations were done. As per ATLS protocol nasal oxygen was started, intravenous access was secured, and blood cross matching was done. He was immediately taken to operating room, and placed in semi left lateral position. Anesthetist intubated him in same position after pre-anesthetic medications and oxygenation, general anesthesia with sevoflurane was administered. First rod impacted in right pectoral area was removed by joining entry and exit wounds of first 12 mm rod. Incision was deepened through right pectoral muscle till retromammary space. There was no injury to ribs, sternum or pleural space was not violated. For removal of second 12 mm rod, incision was taken from entry to exit wound, which when deepened led to laparotomy and mini-thoracotomy. Rod had pierced diaphragm at right costo-phrenic angle and segment six of liver and exited out from segment four of liver. Lung was normal. Gall bladder wad normal. Hepatotomy was done and this rod removed. Hepatotomy was around 15 cm long and 4 cm deep and was closed with no 1 Polyglactin mattress suture thus achieving complete hemostasis. Diaphragm rent of 3 cm × 2.5 cm was closed in two layers with no 1 Polypropylene. Inter-costal drainage tube was placed. There was hemoperitoneum of 100–200 milli-liters only. Abdominal drainage tube was placed. Thorough wash was given and both wounds were closed. Post-operative recovery was uneventful without any wound infection. He was administered anti tetanus serum.

## 3 Discussion

Resuscitation and close monitoring prior to and during surgery are vital with anticipation of major organ and vascular injuries compromising the normal physiology of respiration and circulation. Hypovolemia should be corrected in the OR. Progressive dyspnea can be the most important symptom in patients with penetrating chest injury. The immediate management of thoracic trauma should follow standard advanced trauma life support guidelines [2]. Such type of injured patient should preferably transfer in trauma ambulance to tertiary referral center equipped with trauma center having trauma surgeon, neurosurgeon, cardiovascular surgeon and off course well equipped blood bank. Penetrating injury of abdomen and thorax has been reported in the literature from time to time. Various authors have reported penetration of a number of objects such as glass, knife, wooden blocks, blades, etc. [3]. We encountered penetration of two metallic rods, one penetrating abdomen and thorax and another traversing axilla. Incidence of metallic rod penetration reported in the literature is rare. Such impalement injury is an acute emergency. The anesthetic management of these types of injuries is challenging because of urgency for the surgery and associated hemodynamic instability [4]. Post-operative recovery was uneventful without any wound infection. He was discharged three weeks later with complete wound healing and he resumed his occupation within three months’ time.

## 4 Conclusion

Penetrating abdomino-thoracic injuries demand immediate life-saving measures, appropriate resuscitative care, urgent shifting of patient to tertiary care center, prompt diagnosis and immediate surgical intervention by multi-disciplinary team of abdominal, vascular, and cardiothoracic surgeons [5]. Regulation of safety rules at construction site and early intervention in case of accidents can improve the patient outcome and minimize mortality.

## Sources of funding

No funding.

## Ethical approval

Ethical clearance not taken since it is a single case report. Patient consent obtained.

## Consent

Written informed consent was obtained from the patient for publication of this case report and accompanying images. A copy of the written consent is available for review by the Editor-in-Chief of this journal on request”

## Author contribution

1. Dr. Kushagra Rahul – Assisted for surgery

2. Dr. Aditya Phadke – Assisted for surgery

3. Dr. Sushrut Sankpal – Writing paper.

## Registration of research studies

No research involved.

## Guarantor

Dr Jitendra Sankpal. MS, FACS, FICS, FMAS, FIAGES, FALS, FBMS.

## Provenance and peer review

Editorially reviewed, not externally peer-reviewed

## Declaration of Competing Interest

No conflict of interest with all authors/nothing to disclose for all authors.

## References

[1] Afzal R.M. (2018). Thoracic impalement injury: a survivor with large metallic object in-situ. Chin. J. Traumatol..

[2] Kolahdouzan Mohsen (2016). Impalement thoracoabdominal trauma secondary to falling on metallic (iron) bars: an extremely rare and unique case. Arch. Trauma Res..

[3] Kaur K., Singhal S.K. (2014). Penetrating abdomino-thoracic injury with an iron rod: an anesthetic challenge. Indian Journal of Anesthesia.

[4] Kim Ki Tai, Seo Pil Won (2016). Case of severe thoracoabdominal impalement by a steel bar. Korean J. Thoracic Cardiovasc. Surg..

[5] Liu Y.W., Lee J.Y. (2018). Survival of the fittest: the role of video-assisted thoracoscopic surgery in thoracic impalement injuries. J. Thorac. Dis..

[6] Agha R.A., Borrelli M.R., Farwana R., Koshy K., Fowler A., Orgill D.P., For the SCARE Group (2018). The SCARE 2018 statement: updating consensus surgical CAse REport (SCARE) guidelines. Int. J. Surg..

